# High-throughput detection of parasites and ova in stool using the fully automatic digital feces analyzer, orienter model fa280

**DOI:** 10.1186/s13071-023-06108-1

**Published:** 2024-01-07

**Authors:** Sudarat Boonyong, Saowalak Hunnangkul, Sirirat Vijit, Suphaluck Wattano, Parwin Tantayapirak, Sumas Loymek, Sirichit Wongkamchai

**Affiliations:** 1 Department of Parasitology, Faculty of Medicine Siriraj Hospital, Mahidol University, Bangkok, 10700 Thailand; 2 Department of Research and Development, Faculty of Medicine Siriraj Hospital, Mahidol University, Bangkok, 10700 Thailand; 3 Filaria Project, Phikulthong Royal Development Study Center, Narathiwat, 96000 Thailand

**Keywords:** FA280 autoanalyzer, FECT, Formalin-ethyl acetate, Helminth, Parasitic detection, Protozoa

## Abstract

**Background:**

Intestinal parasitic infections can harm health by causing malnutrition, anemia, impaired growth and cognitive development, and alterations in microbiota composition and immune responses. Therefore, it is crucial to examine stool samples to diagnose parasitic infections. However, the traditional microscopic detection method is time-consuming, labor-intensive, and dependent on the expertise and training of microscopists. Hence, there is a need for a low-complexity, high-throughput, and cost-effective alternative to labor-intensive microscopic examinations.

**Methods:**

This study aimed to compare the performance of a fully automatic digital feces analyzer, Orienter Model FA280 (People’s Republic of China) with that of the formalin-ethyl acetate concentration technique (FECT). We assessed and compared the agreement between the FA280 and the FECT for parasite detection and species identification in stool samples. The first part of the study analyzed 200 fresh stool samples for parasite detection using the FECT and FA280. With the FA280, the automatic feces analyzer performed the testing, and the digital microscope images were uploaded and automatically evaluated using an artificial intelligence (AI) program. Additionally, a skilled medical technologist conducted a user audit of the FA280 findings. The second set of samples comprised 800 preserved stool samples (preserved in 10% formalin). These samples were examined for parasites using the FECT and FA280 with a user audit.

**Results:**

For the first set of stool samples, there was no statistically significant difference in the pairwise agreements between the FECT and the FA280 with a user audit (exact binomial test, *P* = 1). However, there were statistically significant differences between the pairwise agreements for the FECT and the FA280 with the AI report (McNemar’s test, *P* < 0.001). The agreement for the species identification of parasites between the FA280 with AI report and FECT showed fair agreement (overall agreement = 75.5%, kappa [κ] = 0.367, 95% CI 0.248–0.486). On the other hand, the user audit for the FA280 and FECT showed perfect agreement (overall agreement = 100%, κ = 1.00, 95% CI 1.00–1.00). For the second set of samples, the FECT detected significantly more positive samples for parasites than the FA280 with a user audit (McNemar’s test, *P* < 0.001). The disparity in results may be attributed to the FECT using significantly larger stool samples than those used by the FA280. The larger sample size used by the FECT potentially contributed to the higher parasite detection rate. Regarding species identification, there was strong agreement between the FECT and the FA280 with a user audit for helminths (κ = 0.857, 95% CI 0.82–0.894). Similarly, there was perfect agreement for the species identification of protozoa between the FECT and the FA280 with user audit (κ = 1.00, 95% CI 1.00–1.00).

**Conclusions:**

Although the FA280 has advantages in terms of simplicity, shorter performance time, and reduced contamination in the laboratory, there are some limitations to consider. These include a higher cost per sample testing and a lower sensitivity compared to the FECT. However, the FA280 enables rapid, convenient, and safe stool examination of parasitic infections.

**Graphical Abstract:**

**Figure:**
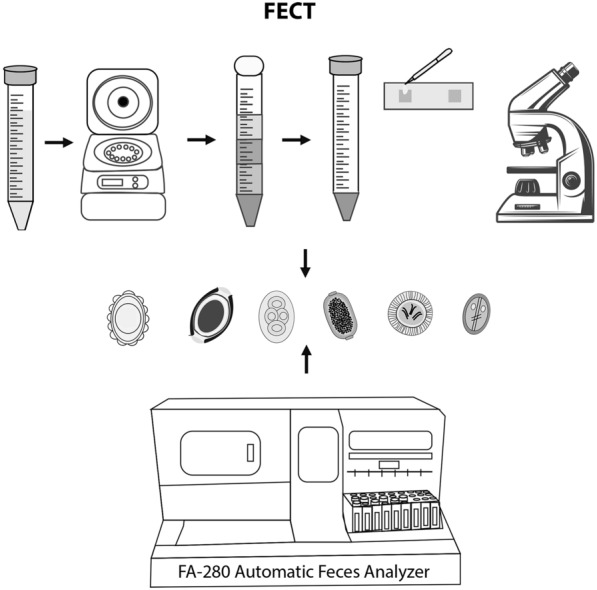

## Background

Parasitic infections pose significant health challenges, particularly in tropical regions. Approximately 3.5 billion people worldwide are affected by intestinal parasites [1, 2]. Intestinal helminth infections can have detrimental effects on health, including malnutrition, anemia, and impaired growth and cognitive development [3–6]. In pregnant women, intestinal helminth infections can lead to inadequate weight gain, intrauterine growth retardation, and low birth weight in newborns [7]. Furthermore, these infections can significantly compromise the mental and educational development of children [3–5, 8]. Protozoa infections are a major contributor to malnutrition, and they may contribute to changes in the microbiota composition and activation of immune responses [2, 9, 10].

Consequently, stool examination is a fundamental tool for diagnosing parasitic infections in clinical laboratories. Macroscopic examination assesses general characteristics such as color, consistency, form, and odor of stool samples, while microscopic examination is used for parasite detection [11]. Various manual methods are available for the detection of intestinal parasites in stool samples, including direct wet smear, Kato’s thick smear, and concentration methods such as simple sedimentation, flotation, and formalin-ethyl acetate [12–14]. Each method has advantages and limitations in terms of sensitivity, stool preparation procedure, and processing time. The spectrum of parasites detected by each method also varies depending on the testing principles and other factors, such as the amount of stool used.

The direct wet smear method is widely used in most clinical laboratories owing to its ease, rapidity, and cost-effectiveness. However, it has been reported to have low sensitivity because of the small stool sample used (0.2 g) [15–18]. Both Kato’s thick smear method and the formalin-ethyl acetate concentration technique (FECT) have demonstrated higher sensitivities than the direct wet smear method [19]. Despite being considered the gold standard for detecting intestinal parasites, microscopic detection is time-consuming, tedious, labor-intensive, and heavily reliant on the expertise and training of microscopists [20]. Therefore, there is a need for low-complexity, high-throughput, and cost-effective tools to replace labor-intensive microscopic examinations.

Several digital imaging-based automated stool examination systems have been developed to diagnose parasitic infections [21–23]. Yang and colleagues were the first to validate an automated stool examination system. They recommended the development of an adjusted algorithm to accurately classify helminth eggs [21]. Subsequently, an automated urine sediment microscopy analyzer, the sediMAX I, was introduced for identification and counting of particles of urine sediment [24]. The sediMAX I microscope has an attachment for a digital camera, with image magnification approximating to 400 × enlargement. All images are analyzed by a high-quality image processing software that is able to detect and classify the particles in urine as blood cells, epithelial cells, crystals, bacteria, yeasts, sperm, and mucus and can be accessed from remote locations [24, 25]. Although the sediMAX I had not yet been approved for detection of intestinal parasites, Indra and colleagues has modified sediMAX-I in detecting intestinal parasites, helminths, and protozoa. They concluded that sediMAX-1 exhibited excellent performance in detecting eggs of *Dibothriocephalus nihonkaiensis*, *Taenia* species, *Hymenolepis nana*, *Trichuris trichiura*, *Ascaris lumbricoides*, and *Paragonimus westermani* [22, 23]. Additionally, FECPAKG2 (Techion, Mosgiel, New Zealand), an image-based diagnostic platform for quantitatively detecting parasite eggs in fecal samples, has also been validated [26–28].

The objective of this study was to compare the efficiency of a fully automatic digital feces analyzer (Model FA280, Orienter, Chengdu, Sichuan, People’s Republic of China) with that of the FECT. The FA280 employs a digital imaging-based method to diagnose parasitic infections in human stool samples. The comparison was based on the following criteria: (1) sensitivity of parasite detection in stool samples, (2) agreement in detecting parasite species in the stool samples, (3) simplicity of the method, (4) practicability of the method for routine diagnosis, and (5) total and hands-on times required to perform the analyses. Through this comparison, we aimed to evaluate the performance and practicality of the digital feces analyzer, thereby assessing its potential as an alternative diagnostic tool for routine parasitic infections.

## Methods

### Study design

**Fig.1:**
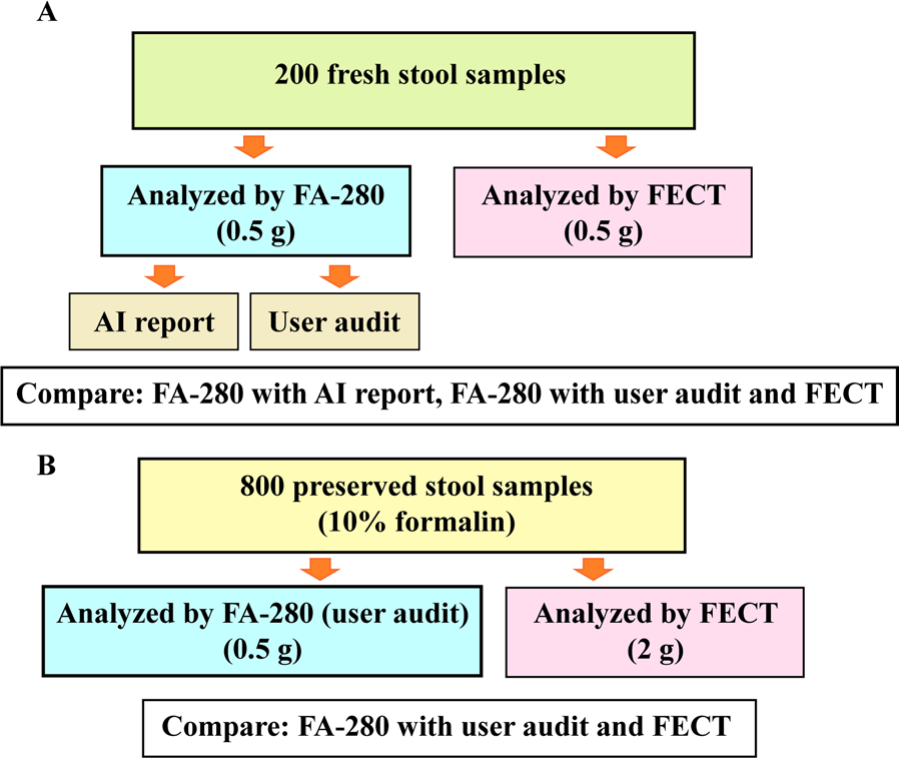
Graphical representation of the study design. The study was divided into two parts: A and B. In Part A of the study, 200 fresh stool samples were analyzed using the Orienter Model FA280 automatic digital feces analyzer. Species identification was evaluated by a skilled medical technologist through user audits, and the results were reported by the analyzer’s artificial intelligence program. In Part B of the study, 800 preserved stool samples were subjected to parasite detection using both the FA280 feces analyzer (with user audit) and the formalin-ethyl acetate concentration technique (FECT). All preserved stool samples were analyzed once with each method

As shown in Fig. 1, two sets of stool samples were utilized in this study. The first set consisted of 200 fresh samples randomly selected from routine stool samples in the Parasitology Laboratory of the Department of Parasitology, Faculty of Medicine Siriraj Hospital, Mahidol University, Thailand. The samples were collected between October and December 2022. A fully automatic digital feces analyzer (Orienter Model FA280) was employed to examine them. The testing results came from an automated report produced by the FA280’s artificial intelligence (AI) program and an auditing report by skilled laboratory technicians. The second set of stool samples comprised 800 samples preserved in 10% formalin. Both the FECT and the FA280, along with a user audit, were used to diagnose parasites in these samples.

### Detection of parasites in stool samples by FECT

The FECT was performed as described by Garcia [29]. In this process, 2 g of stool sample was mixed with 10 ml of 10% formalin. The fecal suspension was then strained through a 2-layer gauze into a 15-ml conical centrifuge tube. To this mixture, 3 ml of ethyl acetate was added. The tube was tightly closed and vigorously shaken in an inverted position for 1 min. The stopper was carefully removed, and the tube was centrifuged at 2500 rpm for 2 min. The plug of debris at the top of the tube was freed by ringing the sides of the tube with an applicator stick. The top layer of the supernatant was decanted, and debris on the sides of the centrifuge tube was removed using a cotton-tipped applicator. The sediment at the bottom of the tube was pipetted onto a clean glass slide, and ova and parasites were observed under a light microscope [29].

### Detection of parasites in stool samples by FA280

**Fig.2:**
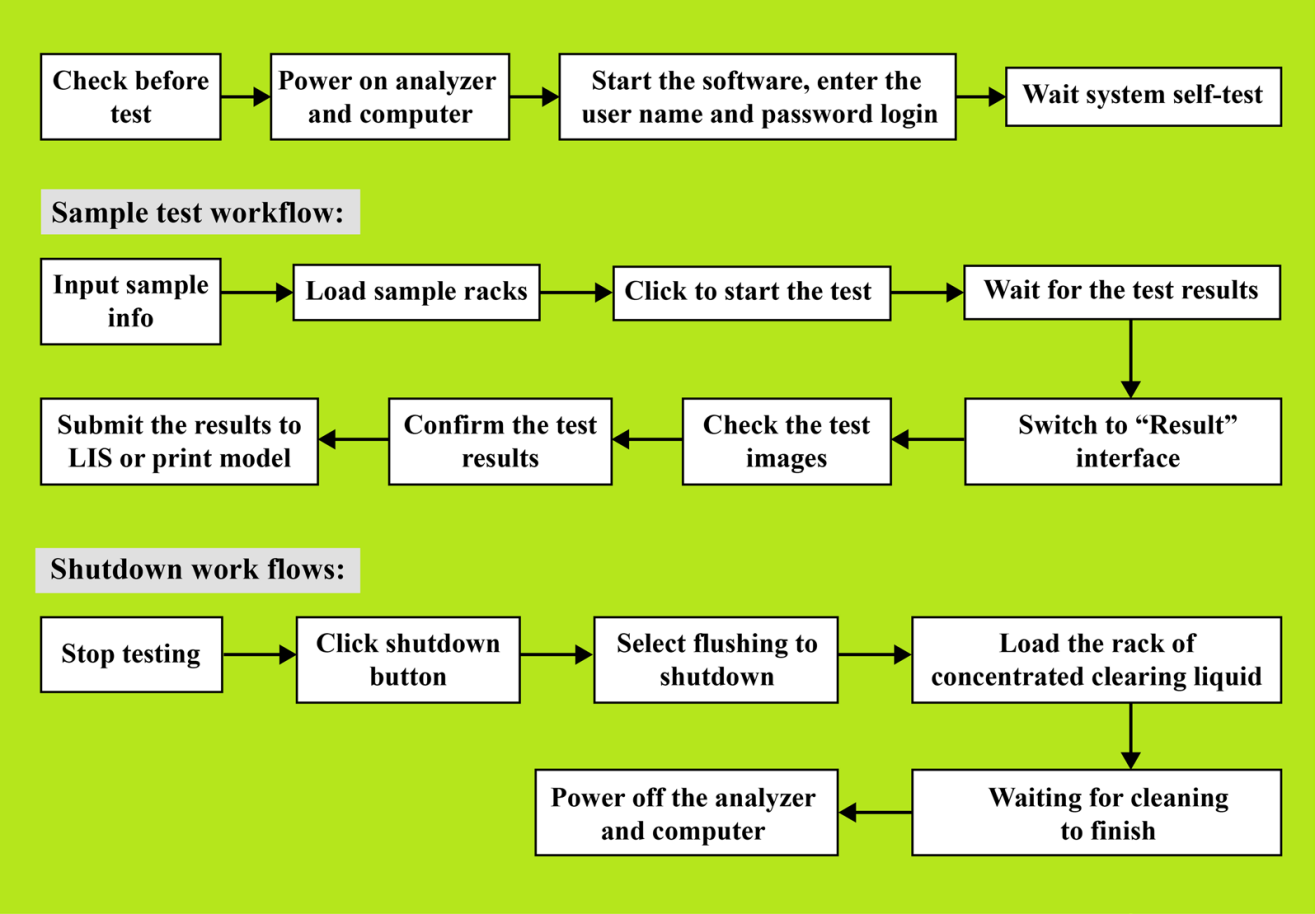
Instrument operation procedures of the FA280 feces analyzer

The FA280 fully automatic digital feces analyzer consists of the following components: (1) automatic in-sample unit. The analyzer uses a track-type sample carrier to ensure accurate and consistent sample loading. (2) Sampling unit. This unit uses a pneumatic mixing system to ensure that the sample is thoroughly mixed with diluent. (3) Sample character and color photographing unit. A high-resolution camera is employed to capture images of the sample. The images are then analyzed by the analyzer’s software to determine the sample’s attributes and color. The results are stored in the system for reference by the tester. (4) Microscope unit. High- and low-power objective lenses are used to automatically capture images of the sample at different magnifications. The microscope unit also utilizes multifield tomography imaging to obtain detailed sectional images of the sample. (5) Test kit unit. This unit facilitates the quantification of samples by dropping them into the sample loading area of the test kit. The test kit incubation belt automatically rotates the test kit to the image collection area to periodically capture and interpret photos of the experimental reaction results. Illustrations detailing the operational procedures of the FA280 analyzer are shown in Fig. 2.

The test principle of the FA280 analyzer is based on a simple sedimentation technique. In the processing step, a batch of 40 stool samples is processed in a single run, which takes approximately 30 min. During sample testing, approximately 0.5 g of a stool sample is placed in a filtered sample collection tube using a spoon. The sample collection tube is then placed into the sample rack of the analyzer.

Once the system obtains a sample, the software initiates the test. Microscopic examination is performed, capturing photographs of the stool sample and recording its various attributes. These attributes encompass the sample’s color, form, and consistency. The color varies from yellow, off-white, green, and red to a tar-like hue. The form refers to the physical shape or appearance, while the consistency indicates whether the sample is loose, semisolid, or watery.

Next, a diluent is automatically added to the sample in the tube. The main components of the diluent typically include sodium chloride (approximately 0.85–0.95%), PC-300 (approximately 0.1–0.3 ml/l), and water for analytical laboratory use. The diluent is mixed thoroughly with the stool sample through pneumatic mixing. This process helps filter out nonpathological residues such as large plant fibers, seeds, and undigested residual food. The prepared sample enters a counting cell within the analyzer’s sample character and color photographing unit. The microscope unit of the FA280 automatically focuses and collects high-resolution images of the sample.

In our investigation, the digital microscope images were uploaded and analyzed using the system software, which generated an AI report. Additionally, an experienced medical technologist performed a user audit by reviewing the uploaded images on screen to check for the presence of parasites.

### Statistical analysis

Statistical analyses were conducted using GraphPad Prism version 7.04 (GraphPad Software Inc, San Diego, CA, USA). Differences in parasite-positive samples detected by the FA280 analyzer (both through the AI report and the user audit) and the FECT were evaluated using McNemar’s test. The consistency in parasite species detection between techniques was assessed via kappa (κ) agreement, with a 95% confidence interval (95% CI).

## Results

### Comparison of the FECT and the FA280 for parasite detection

For the first set of stool samples, Table 1 presents the results of parasite detection in 200 fresh stool samples tested using the FECT, FA280 with an AI report, and FA280 with a user audit. The pairwise agreements between the results obtained with the FECT and the FA280 with an AI report showed statistically significant differences (McNemar’s test, *P* < 0.001). However, there were no statistically significant differences between the results obtained from the FECT and FA280 with a user audit (exact binomial test, *P* = 1.000). When comparing the agreement for the species of parasites detected by the FA280 with an AI report, fair agreement was observed (overall agreement 75.5% [151/200 samples], κ = 0.367, 95% CI 0.248–0.486). On the other hand, the agreement for the species of parasites detected by the FA280 with a user audit and FECT showed perfect agreement (overall agreement 100%, κ = 1.00, 95% CI 1.00–1.00).

**Table 1:** Results of parasite detection of 200 fresh stool samples obtained from routine parasite detection using the formalin ethyl acetate concentration technique (FECT)

Method	Negative	Positive	Species of parasite detected						
			Al	Tt	Hw	Ts	Ov	Bh	Gl
microscopic detection (FECT)	178	22	11	6	1	0	0	3	1
FA-280 feces analyzer (AI report)	129	71	13	6	8	14	41	0	2
FA-280 feces analyzer (user audit)	178	22	11	6	1	0	0	3	1

*Al* *Ascaris lumbricoides*, *Tt* *Trichuris trichiura*, *Hw* hookworm, *Ts* *Taenia* spp., *Ov* *Opisthorchis viverini*, *Bh* *Blastocystis hominis*, *Gi* *Giardia lamblia*

The FA280 with an AI report demonstrated a substantial false-positive rate of 32.5% (65/200 samples). In light of this high rate, the performance of the FECT was compared to that of the FA280 with a user audit in the second set of stool samples.

For the second set of stool samples, as presented in Table 2, parasites were detected in 468 of the 800 samples tested by the FECT, while the FA280 with a user audit detected parasites in 440 of the samples. The results indicated that the FECT identified a significantly higher number of parasite-positive samples than the FA280 with a user audit (McNemar’s test, *P* < 0.001). Table 3 compares the performance of three methods: direct wet smear, the FECT, and the FA280 with a user audit.

**Table 2:** Results of parasite detection of 800 preserved stool samples using FA-280 feces analyzer (user audit) and the formalin ethyl acetate concentration technique (FECT)

Methods	Number of stool sample	
	Positive	Negative
Formalin ethyl acetate concentration (FECT)	468	332
FA280 feces analyzer (user audit)	440	360

^*^Agreement for all parasite positive samples detected between the autoanalyzer using result obtained from user audit and FECT revealed weighted kappa (κ) values of 0.857, indicating almost perfect agreement

**Table 3:** Comparison of the parasite detection performance of three methods: direct wet smear, formalin-ethyl acetate concentration technique (FECT), and FA280 feces analyzer (user audit)

Parameter compared	Direct wet smear [29]	FECT	FA280 with user audit
Weight of stool used	0.2 g	2 g	0.5–1 g
Technique	Manual	Manual	Automatic
Process simplicity	Less complicated	More complicated	Less complicated
Processing time	2 min/sample	8–10 min/sample	2 min/sample
Parasite observing time	5–10 min	5–10 min	3–5 min
Parasites observation tool	Microscope	Microscope	High-resolution images
Experienced laboratory technician	Required	Required	Required
Result recorded and stored	No	No	Yes
Cost/test	USD 0.25	USD 0.50	USD 2.00

**Fig. 3:**
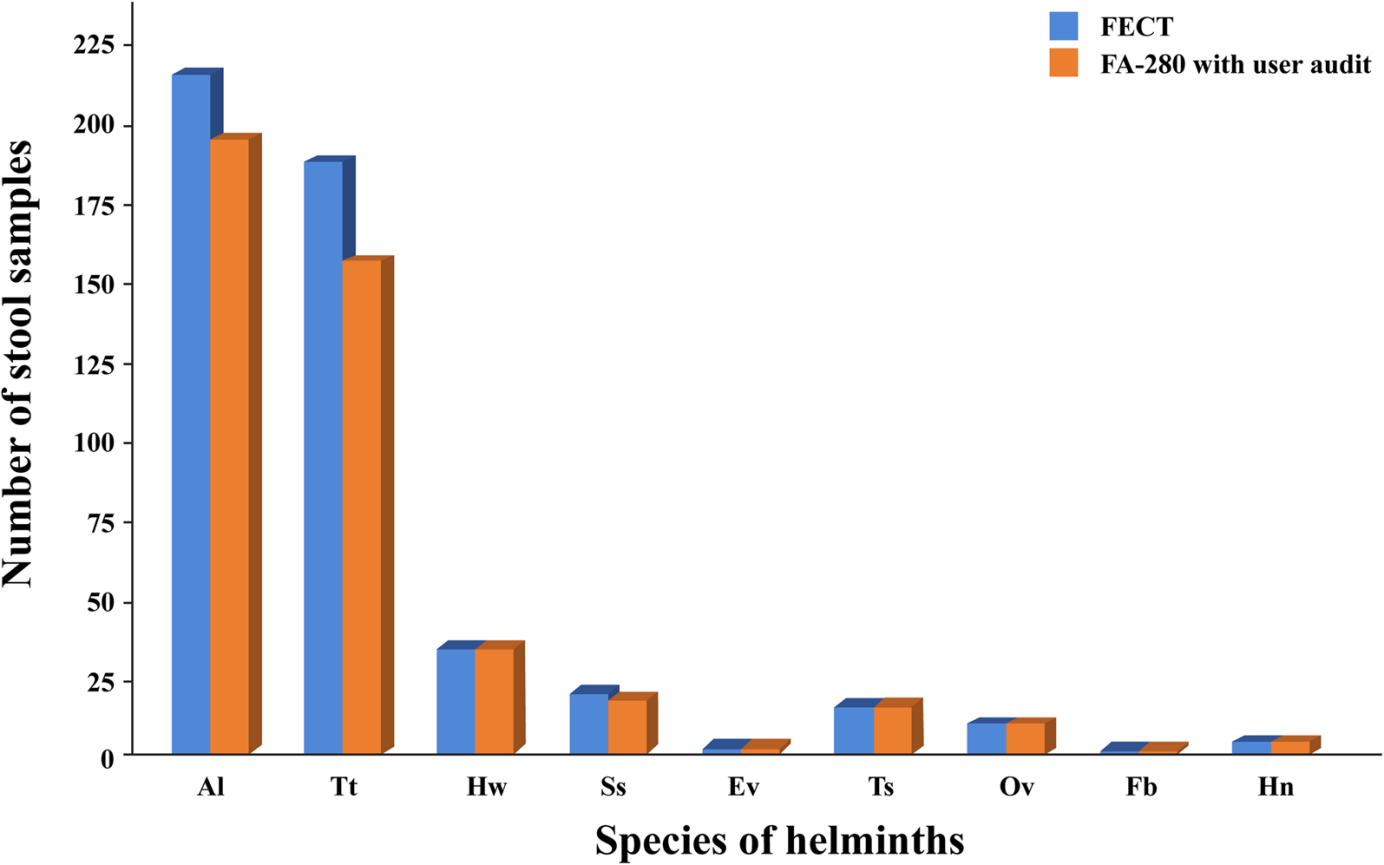
Bar graphs showing the number and helminth species found in the test stool samples tested by the formalin-ethyl acetate concentration technique (FECT) and the FA280 feces analyzer (with user audit)

**Fig. 4:**
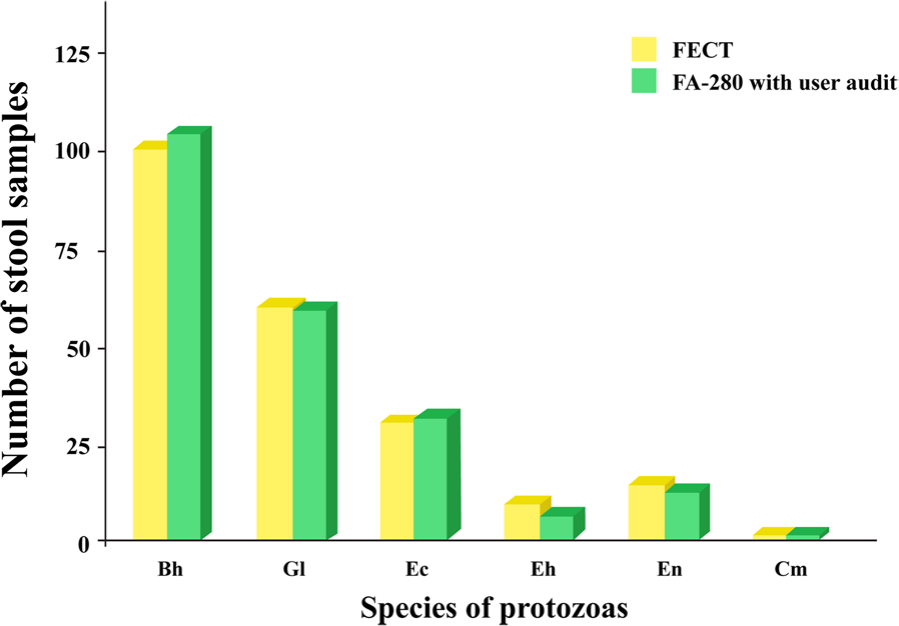
Bar graphs showing the number and protozoa species found in the test stool samples tested by the formalin-ethyl acetate concentration technique (FECT) and the FA280 feces analyzer (with user audit)

**Fig. 5:**
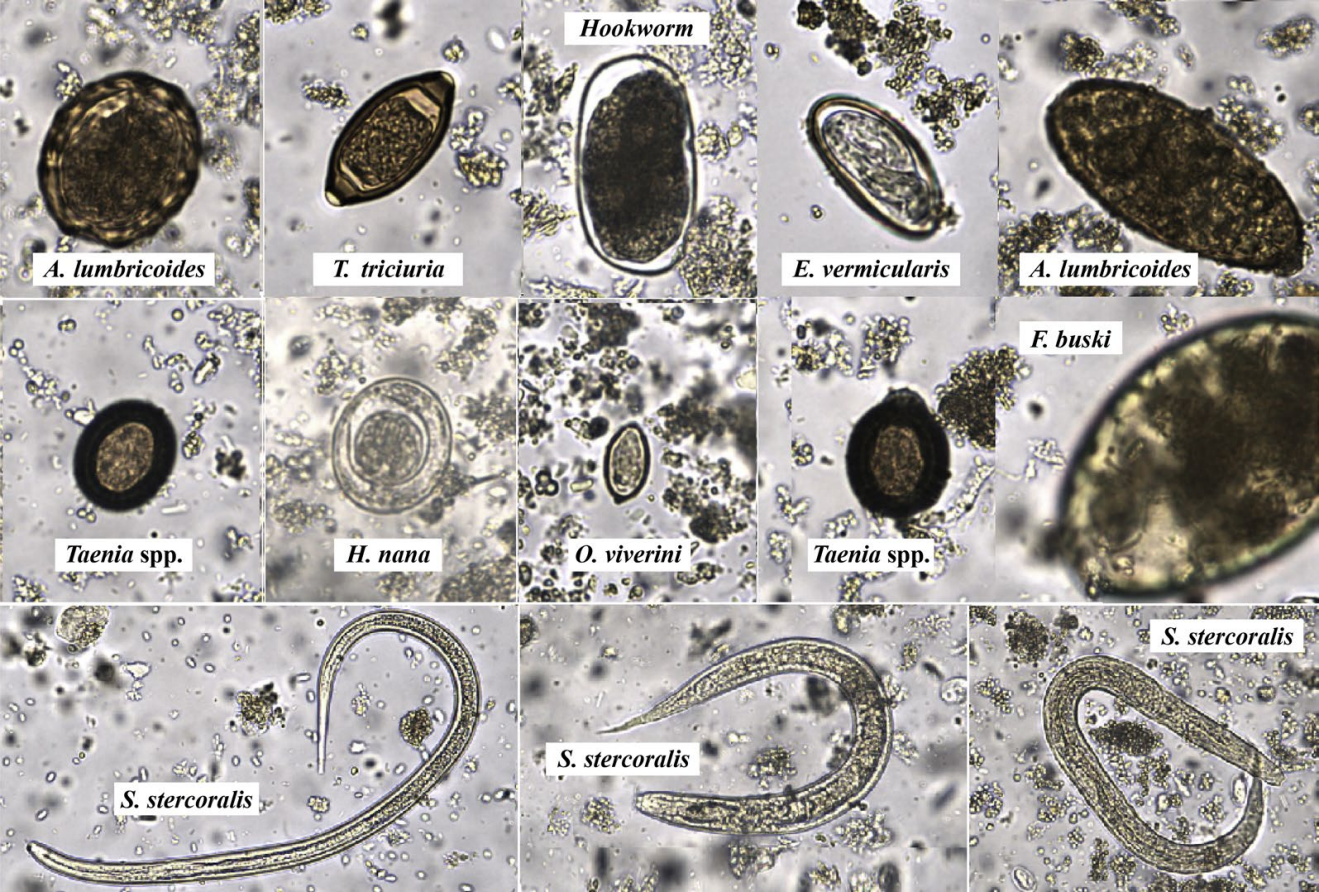
High-resolution images from the FA280 feces analyzer’s digital microscope depicting helminth eggs and larvae

**Fig. 6:**
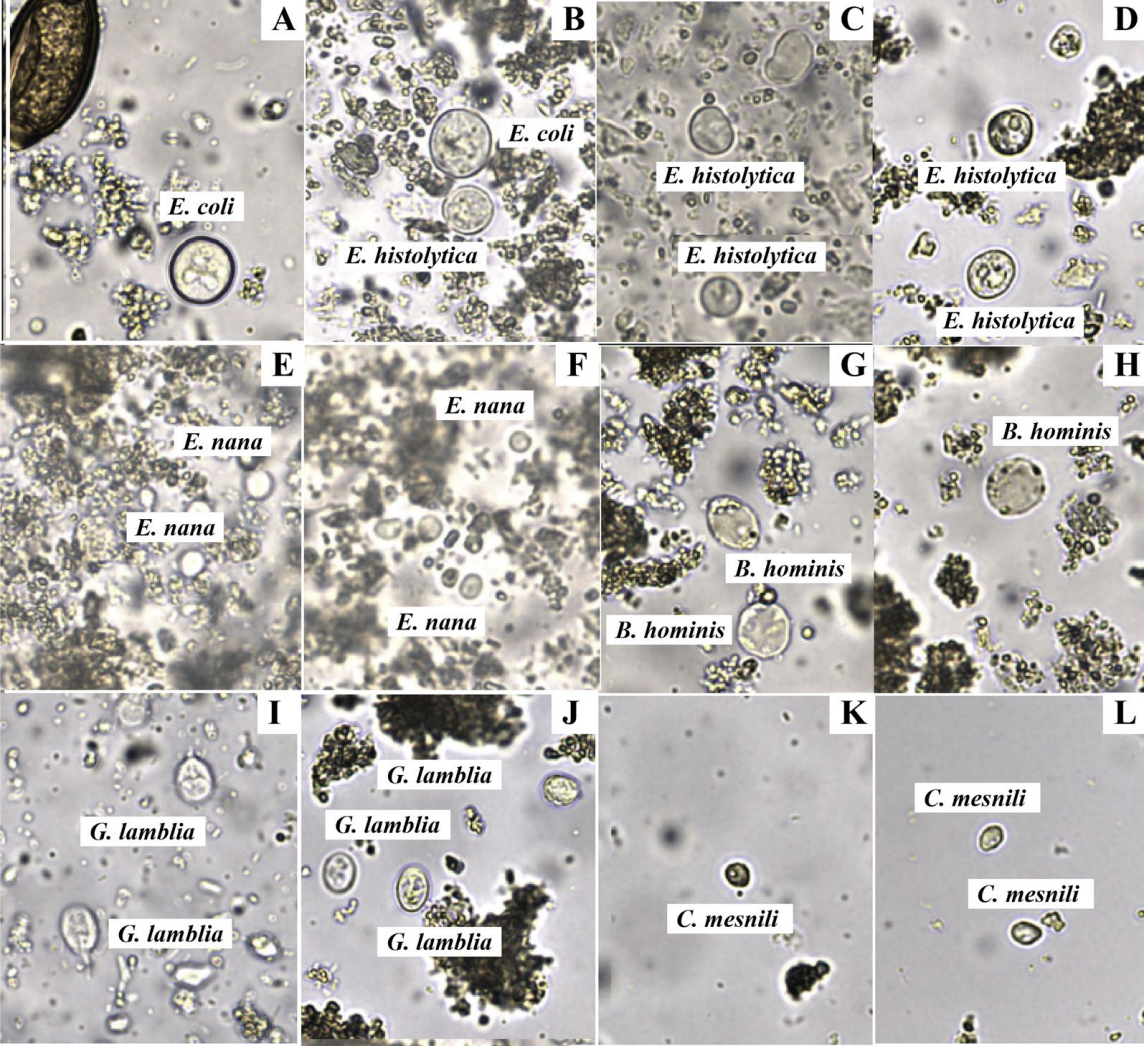
High-resolution images from the FA280 feces analyzer’s digital microscope depicting protozoan cysts and trophozoites

Figures 3 and 4 display bar graphs illustrating the species of helminths and protozoa, respectively, detected by the FECT and the FA280 with a user audit. Figure 5 shows high-resolution images from the FA280 digital microscope depicting helminth eggs and larvae. Additionally, Fig. 6 shows images of protozoan cysts and trophozoites. The concordance in detecting helminth species between the FECT and the FA280 (when used with a user audit) was notably strong. Of the 800 samples, 747 were consistent, yielding an overall match rate of 93.4%. The kappa value was 0.857, with a 95% confidence interval of 0.82–0.894. Similarly, the concordance in detecting protozoan species between the FECT and the FA280 (when used with a user audit) was perfect. All 800 samples matched, resulting in a 100% consistency rate (κ = 1.00, 95% CI 1.00–1.00).

## Discussion

Our study compared the performance of the FECT, the FA280 with an AI report, and the FA280 with a user audit in diagnosing parasitic infections in 1000 stool samples. The study’s strengths were as follows: using the same stool samples to evaluate the performance of all techniques, employing a large number of fresh and preserved stool samples (1000), having a substantial number of parasite-positive samples (468), and encompassing a variety of helminth and protozoan species.

Our findings revealed that the FECT identified more positive samples than the FA280 with a user audit (468 vs. 440). Both the FECT and the FA280 analyzer utilize the sedimentation method. It is considered the most straightforward and least error-prone technique, ensuring the recovery of helminth eggs, helminth larvae, and protozoan cysts [29]. However, several factors may explain the discrepancy between the number of positive results found by the FECT and the FA280 with a user audit. First, the FECT requires 2 g of stool sample, whereas the FA280 analyzer uses 0.5 g. The larger stool sample for the FECT contributes to a higher parasite detection rate. Second, the processing steps in the FECT, including the removal of fecal debris and fat from stool samples using ethyl acetate, enhance egg isolation, thereby increasing the chances of detecting parasite eggs or larvae. Regarding the species of detected parasites, the FA280 with the user audit demonstrated strong to perfect agreement with the FECT in detecting intestinal helminth and protozoan species (κ = 0.857 and 1, respectively).

This study also demonstrated that using fresh stool samples resulted in perfect agreement with the results obtained from the FECT, whereas the testing with the preserved stool samples yielded a lower positive-sample detection rate. This discrepancy can be explained by the fact that formalin-preserved stool samples have an exact stool weight of < 0.5 g due to their dilution with 10% formalin. Additionally, the first set of fresh stool samples consisted of only 200 samples with 22 positive parasite samples, compared to the 800 preserved stool samples with 468 positive parasite samples. Sample size plays a crucial role in decision-making analysis: the larger the sample used, the more reliable the results are.

The FECT also detected more *A. lumbricoides* and *T. trichiura* positive-stool samples than the FA280 with a user audit. This discrepancy may be attributed to the worm burden in infected individuals. In some instances where the FECT detected *A. lumbricoides*- or *T. trichiura*-positive samples but the FA280 analyzer yielded negative results, only a few or rare parasite eggs were observed under the microscope. Stool samples with a high abundance of parasite eggs can increase detection rates in microscopy-based examinations [30]. Thus, in stool samples with low density of the parasites eggs, along with the lower amount of the stool samples usage, might have led to the lower detection rate of FA-280. Additionally, this discrepancy in results may be explained by the study of Brummaier and co-authors who found that the FECT was superior in detecting hookworm and *T. trichiura* eggs [30].

Although the FECT has several advantages, there are some precautions to consider. The processing steps of the FECT are time-consuming, including the shaking step before centrifugation and removal of debris plugs, which must be performed carefully for accurate results. Moreover, processing many stool samples is tedious and laborious. Observing large amounts of stool sediment under the microscope often causes eye fatigue and dizziness in operators.

The FA280 analyzer offers several advantages. First, as the testing and analysis are fully automated, processing times and human errors are reduced, making the analyzer suitable for the mass screening of parasitic infections. Second, the stool preparation process is carried out in a closed system, ensuring the safety of laboratory technicians and reducing potential contamination in the laboratory. Third, stool sediments can be stored for several weeks, allowing for retesting or utilization for education or research. Finally, using a digital microscope, the FA280 analyzer records high-resolution images of the detected parasites. These images can be retrospectively observed and used for educational and quality control purposes.

However, the present study raises some concerns regarding the application of automatic feces analyzers for parasitic detection. In routine laboratory diagnosis of parasitic infections, microscopic-based methods are commonly used. Laboratory technicians are familiar with the appearance of parasite eggs, larvae, cysts, and trophozoites observed under a microscope. In contrast, the FA280 is an image-based diagnostic platform that utilizes high-resolution digital microscopic images for evaluation and data collection. From our experience, the high-resolution images obtained from the FA280 may differ somewhat from those observed under a microscope, potentially leading to the misdiagnosis of parasites. Therefore, before implementing the diagnosis of parasitic infections using an FA280 analyzer or a similar image-based diagnostic platform, laboratory technicians should train extensively by reviewing digital images of parasites to ensure familiarity.

Furthermore, the FA280 with AI automatic reporting showed limited analytical specificity in the present study. Therefore, the FA280 automatic feces analyzer cannot fully replace a skilled operator. It still requires an experienced or well-trained laboratory technician to evaluate the digital microscope images to obtain accurate results and avoid misdiagnosing parasitic infections. Additionally, the FA280, equipped with a special type of microscope and an electronic camera, allows for clear visualization of the inner contents of cysts, particularly in *Giardia duodenalis*. However, for cysts of other amoebas, such as *Entomoeba coli* and *E. histolytica/dispar*, the size of the cyst is another key factor used to differentiate between species within the *Entamoeba* genus.

## Conclusions

This study assessed and compared the concordance in detecting parasites and identifying species in stool samples using a fully automatic digital feces analyzer (Orienter Model FA280) and the FECT. Despite the higher cost per sample and lower sensitivity of the FA280 compared to the FECT, the FA280 automatic feces analyzer offers the advantage of high automation, simplified operation procedures, rapid detection speed, and an improved working environment.

## Data Availability

All data generated or analyzed during this study are included in the published article.

## Abbreviations

FECT: Formalin ethyl acetate concentration technique
AI: Artificial intelligence
G: Gram

## References

[1] Shiferaw K, Tesfay T, Kalayu G, Kiros G (2021). Human intestinal parasites: prevalence and associated risk factors among grade school children in maksegnit. Northwest Ethiopia J Trop Med.

[2] Wattano S, Kerdpunya K, Keawphanuk P, Hunnangkul S, Loimak S, Tungtrongchitra A (2023). An epidemiological survey of intestinal parasitic infection and the socioeconomic status of the ethnic minority people of Moken and orang Laut. Trop Med Infect Dis.

[3] Oninla SO, Onayade AA, Owa JA (2010). Impact of intestinal helminthiases on the nutritional status of primary-school children in Osun state, south-western Nigeria. Ann Trop Med Parasitol.

[4] Fürst T, Silué KD, Ouattara M, N’Goran DN, Adiossan LG, N’Guessan Y (2012). Schistosomiasis, soil-transmitted helminthiasis, and sociodemographic factors influence quality of life of adults in Côte d’Ivoire. PLOS Negl Trop Dis.

[5] Sayasone S, Utzinger J, Akkhavong K, Odermatt P (2015). Multiparasitism and intensity of helminth infections in relation to symptoms and nutritional status among children: a cross-sectional study in southern Lao People’s Democratic Republic. Acta Trop.

[6] Ellwanger JH, Ziliotto M, Kulmann-Leal B, Chies JAB (2022). Iron deficiency and soil-transmitted helminth infection: classic and neglected connections. Parasitol Res.

[7] Rodríguez-Morales A, Barbella RA, Case C, Arria M, Ravelo M, Perez H (2006). Intestinal parasitic infections among pregnant women in venezuela. Infect Dis Obstet Gynecol.

[8] Fink MY, Singer SM (2017). The intersection of immune responses, microbiota and pathogenesis in giardiasis. Trends Parasitol.

[9] Hotez PJ, Alvarado M, Basáñez MG, Bolliger I, Bourne R, Boussinesq M (2014). The global burden of disease study 2010: interpretation and implications for the neglected tropical diseases. PLoS Negl Trop Dis.

[10] Bouree P, Bisaro F (2007). Parasitic diarrhea. Presse Med.

[11] Kasırga E (2019). The importance of stool tests in diagnosis and follow-up of gastrointestinal disorders in children. Turk Pediatri Ars.

[12] Koontz F, Weinstock JV (1996). The approach to stool examination for parasites. Gastroenterol Clin N Am.

[13] Demeke G, Fenta A, Dilnessa T (2021). Evaluation of wet mount and concentration techniques of stool examination for intestinal parasites identification at Debre Markos comprehensive specialized hospital. Ethiopia Infect Drug Resist.

[14] Labpedia net. 2021. Stool examination: stool smear preparation, stains, handling, and preservatives. 2021. https://www.labpedia.net/stool-examination-part-3-stool-smear-preparation-stains-handling-and-preservatives/. Accessed 30 July 2023.

[15] Endris M, Tekeste Z, Lemma W, Kassu A (2013). Comparison of the Kato-Katz, wet mount, and formol-ether concentration diagnostic techniques for intestinal helminth infections in Ethiopia. ISRN Parasitol.

[16] Amin HA, Ali SA (2015). Evaluation of different techniques of stool examination for intestinal parasitic infections in Sulaimani City-Iraq. Int J Curr Microbiol App Sci.

[17] Hailu T, Abera B (2015). Performance evaluation of direct saline stool microscopy, formol ether concentration and Kato Katz diagnostic methods for intestinal parasitosis in the absence of gold standard methods. Trop Dr.

[18] Yimer M, Hailu T, Mulu W, Abera B (2015). Evaluation performance of diagnostic methods of intestinal parasitosis in school age children in Ethiopia. BMC Res Notes.

[19] Aiadsakun P, Sriwimol W, Thongbun N, Rui-On B, Thiparaksaphan K, Phainuice C (2022). Comparison of the complete filtration method using an automated feces analyzer with three manual methods for stool examinations. J Microbiol Methods.

[20] Han S, Zhang X, Wen J, Li Y, Shu J, Ling H (2012). A combination of the Kato-Katz methods and ELISA to improve the diagnosis of clonorchiasis in an endemic area. China PLoS One.

[21] Yang YS, Park DK, Kim HC, Choi MH, Chai JY (2001). Automatic identification of human helminth eggs on microscopic fecal specimens using digital image processing and an artificial neural network. IEEE Trans Biomed Eng.

[22] Intra J, Taverna E, Sala MR, Falbo R, Cappellini F, Brambilla P (2016). Detection of intestinal parasites by use of the cuvette-based automated microscopy analyser sediMAX®. Clin Microbiol Infect.

[23] Intra J, Sala MR, Falbo R, Cappellini F, Brambilla P (2017). Improvement in the detection of enteric protozoa from clinical stool samples using the automated urine sediment analyzer sediMAX® 2 compared to sediMAX® 1. Eur J Clin Microbiol Infect Dis.

[24] Zaman Z, Fogazzi GB, Garigali G, Croci MD, Bayer G, Kranicz T (2010). Urine sediment analysis: analytical and diagnostic performance of sediMAX®—a new automated microscopy image-based urine sediment analyzer. Clin Chim Acta.

[25] Falbo R, Sala MR, Signorelli S, Venturi N, Signorini S, Brambilla P (2012). Bacteriuria screening by automated whole-field-image-based microscopy reduces the number of necessary urine cultures. J Clin Microbiol.

[26] Boelow H, Krücken J, Thomas E, Mirams G, Samson-Himmelstjerna G (2022). Comparison of FECPAKG2, a modified Mini-FLOTAC technique and combined sedimentation and flotation for the coproscopic examination of helminth eggs in horses. Parasit Vectors.

[27] Tyson F, Dalesman S, Brophy PM, Morphew RM (2020). Novel equine faecal egg diagnostics: validation of the FECPAKG2. Animals.

[28] Rashid MH, Stevenson MA, Waenga S, Mirams G, Campbell AJD, Vaughan JL (2018). Comparison of McMaster and FECPAK(G2) methods for counting nematode eggs in the faeces of alpacas. Parasit Vectors.

[29] Garcia LS (2001). Diagnostic medical parasitology.

[30] Brummaier T, Archasuksan L, Watthanakulpanich D, Paris DH, Utzinger J, McGready R (2021). Improved detection of intestinal helminth infections with a formalin ethyl-acetate-based concentration technique compared to a crude formalin concentration technique. Trop Med Infect Dis.

